# Do practice characteristics explain differences in morbidity estimates between electronic health record based general practice registration networks?

**DOI:** 10.1186/s12875-014-0176-7

**Published:** 2014-10-30

**Authors:** C van den Dungen, N Hoeymans, M van den Akker, MCJ Biermans, K van Boven, JHK Joosten, RA Verheij, MWM de Waal, FG Schellevis, JAM van Oers

**Affiliations:** Department Tranzo, Faculty of Social and Behavioural Sciences, Tilburg University, PO Box 90153, 5000 LE Tilburg, the Netherlands; National Institute for Public Health and the Environment (RIVM), Bilthoven, the Netherlands; Department of Family Medicine, School for Public Health and Primary Care (Caphri), Maastricht University, Maastricht, the Netherlands; Department of General Practice, KU Leuven, Leuven, Belgium; Department of Primary and Community Care, Radboud University Nijmegen Medical Centre, Nijmegen, the Netherlands; General Practitioner, Franeker, the Netherlands; Department of General Practice and Elderly Care Medicine, The Academic Network of General Practitioners of the VU University, VU University Medical Centre, Amsterdam, the Netherlands; Netherlands Institute for Health Services Research (NIVEL), Utrecht, the Netherlands; Department of Public Health and Primary Care, Leiden University Medical Center, Leiden, the Netherlands; Department of General Practice & Elderly Care Medicine/EMGO Institute for health and care research, VU University Medical Centre, Amsterdam, the Netherlands

**Keywords:** Family practice, Incidence, Electronic medical records, Practice characteristics, Population health, Prevalence

## Abstract

**Background:**

General practice based registration networks (GPRNs) provide information on population health derived from electronic health records (EHR). Morbidity estimates from different GPRNs reveal considerable, unexplained differences. Previous research showed that population characteristics could not explain this variation. In this study we investigate the influence of practice characteristics on the variation in incidence and prevalence figures between general practices and between GPRNs.

**Methods:**

We analyzed the influence of eight practice characteristics, such as type of practice, percentage female general practitioners, and employment of a practice nurse, on the variation in morbidity estimates of twelve diseases between six Dutch GPRNs. We used multilevel logistic regression analysis and expressed the variation between practices and GPRNs in median odds ratios (MOR). Furthermore, we analyzed the influence of type of EHR software package and province within one large national GPRN.

**Results:**

Hardly any practice characteristic showed an effect on morbidity estimates. Adjusting for the practice characteristics did also not alter the variation between practices or between GPRNs, as MORs remained stable. The EHR software package ‘Medicom’ and the province ‘Groningen’ showed significant effects on the prevalence figures of several diseases, but this hardly diminished the variation between practices.

**Conclusion:**

Practice characteristics do not explain the differences in morbidity estimates between GPRNs.

**Electronic supplementary material:**

The online version of this article (doi:10.1186/s12875-014-0176-7) contains supplementary material, which is available to authorized users.

## Background

In the Netherlands, routinely collected data from general practice based registration networks (GPRNs) are often used to monitor incidence and prevalence of diseases in the general population. The Dutch Public Health Status and Forecasts 2010, for example, showed the morbidity figures of several diseases using such data [1,2].

This data derived from electronic health records (EHR) in general practice is relevant because the general practitioner (GP) is gatekeeper to secondary care and nearly all inhabitants are enlisted to a single GP (list system). Therefore, GPs have contact with a variety of patients, regarding age, gender, socio-economic status, ethnicity, health problems and disease stage. Furthermore, the list system makes a precise determination of the epidemiological denominator possible [3,4].

The estimated incidence and prevalence figures of different diseases show considerable variations between GPRNs. These differences in morbidity estimates are not fully understood, making the interpretation of these figures difficult [4,5]. The prevalence of osteoarthritis, for example, ranges from 10 to 60 per 1000 person years between GPRNs. Overall, prevalence figures estimated from GPRNs show more variation than incidence Figures [6].

A generally recognized reason for variation in incidence and prevalence figures is the differences in practice population characteristics. For example, the prevalence of osteoarthritis is higher in older people, leading to a higher estimate of the prevalence of this disease in GPRNs with a higher proportion of elderly people in their practice populations. However, in previous research, we showed that population characteristics could not explain the variation between GPRNs or between general practices [6].

In earlier research, we identified the GP and practice characteristics as a probable factor of variation between morbidity estimates using GPRN data, also known as inter-doctor variation [5]. Inter-doctor variation is the variation in the frequency of diagnosing health problems between different health care providers, which cannot be explained by the patient characteristics (age, sex, severity of the disease) [7]. Research identified different aspects that influence this inter-doctor variation between GPs and practices [8-10]. Examples of such characteristics are availability of health care, organization of care, such as type of practice, employment of a practice nurse and treatment opportunities [8,9]. There is evidence that different doctor- and practice characteristics, such as experience, workforce, and type of practice, influence medical practice and diagnostic variability. In the second Dutch National Survey of General Practice, urbanization level, type of practice and EHR software package influenced consultation frequency Figures [10].

In this paper we investigate to what extent practice and GP characteristics explain the variation in morbidity estimates between six Dutch GPRNs and related practices.

## Method

### Databases

Six Dutch GPRNs participated in this research; the Continuous Morbidity Registration Nijmegen (CMR-N), the Academic Network of General Practitioners of the VUmc (ANH-VUmc), the Netherlands Information Network of General Practice (LINH)^a^, the Registration Network of General Practitioners Associated with Leiden University (RNUH-LEO), the Study of Medical Information and Lifestyle in Eindhoven (SMILE) and the Transition project (Trans). More detailed information of these GPRNs can be found elsewhere [5]. These Dutch GPRNs were selected, because they collect information on all health problems of individual patients. GPRNs which exclusively collect information on chronic, permanent or recurring diseases were left out of this study.

### Using the data

We performed an observational study without any intervention. In the Netherlands, no approval is necessary from an ethical committee for analysing data from general practice registration networks. The data are not openly available, permission to use the data is granted by ANH VUmc, RNUH_LEO, SMILE, Transition project, LINH steering committee and the chair of CMR-N.

### Selection of diseases

For our analyses we selected twelve health problems: urinary tract infection, gastro-intestinal infection, neck and back problems, eczema, asthma, chronic obstructive pulmonary disease (COPD), osteoarthritis, diabetes mellitus, coronary heart disease (CHD), stroke, depression, and anxiety disorders. The selection of these health problems was based on three criteria: (1) The expected incidence of the specific disorder in the Dutch general practice population was at least 3 per 1000 per year; (2) The total set of diseases represented several ICD chapters (e.g. circulatory system, respiratory system) to obtain a broad spectrum of diseases; (3) The occurrence of incidence and prevalence of included diseases should vary between different patient subgroups (e.g. age, gender).

### Incidence and prevalence rates

In this study, we used data of 2007. To determine incidence rates, all patients diagnosed with a new episode of a certain disease between the 1st of January 2007 and the 31st of December 2007 were counted per 1,000 patient years. Prevalence rates were calculated by counting the number of patients with a new or existing episode of a specific disease in 2007 per 1,000 patient years (period prevalence). Incidence rates were calculated for all twelve diseases; prevalence rates were only calculated for the ten chronic or recurring diseases. Five GPRNs record diagnoses according to the International Classification of Primary Care (ICPC), one used the so-called E-list codes [11-13]. When necessary, we combined different classification codes to determine morbidity [1,14]. For example, to measure depression we used ICPC codes P03 and P76.

### Socio-demographic characteristics

This study starts with analysing the variation in incidence and prevalence figures between GPRNs and general practices adjusted for patient characteristics: age (in years), gender (male versus female), socio-economic status (high-medium-low), urbanization level (‘rural’, ‘urban’ and ‘large cities’) and ethnic origin [6]. The latter three measures were determined by proxy using 4-digit postal codes of the patients’ home address (the population size is about 4,000 per postal code area) [15,16].

### Practice characteristics

Within a general practice, patients are generally registered with one specific GP, but most patients are not exclusively treated by that GP. The care in general practice has become more multi-practitioner and multi-disciplinary organized [3]. In most networks, the information of an individual patient cannot be related to an individual doctor and therefore, inter-doctor variation cannot be assessed validly. Instead, we analysed GP characteristics on practice level.

The practice characteristics used in the analysis are type of practice (one GP = solo, two GPs = duo and three GPs or more = group practice), percentage female GPs, mean years of working experience, employment of a practice nurse (yes/no), EHR software package used in the general practice, province, distance to the nearest out-of-hours service location and distance to the nearest hospital.

To be sure all practice characteristics are based on the same type of data we consulted the “Register of General Practitioners” (HAREG) of NIVEL [17]. This database holds information on all practising GPs and practices in the Netherlands about e.g. gender, age, and working experience. We received the information about the employment of a practice nurse and type of electronic patient record directly from the GPRN. The distances have been calculated with the so-called driving time model of Automotive Navigation Data (AND) in combination with the localisations of the out-of-hours service locations and hospitals, using 4-digit postal codes [18].

We are interested in the influence of practice characteristics on the variation in morbidity estimates between GPRNs and practices. Therefore, we only used the practices with all population and practice characteristics available. As a consequence, 9 out of 81 practices of LINH, 2 out of 9 practices of ANH VUmc and 1 out of 9 practices of SMILE and 1 out of 5 practices of Trans were excluded from analyses.

### Analyses

Descriptive analyses were applied to give an overview of the distribution of the population and practice characteristics. To explore the variation in morbidity estimates between GPRNs and general practices we used multilevel logistic regression analysis with three levels (patient, practice and network). We used random intercepts on network and practice level to determine the unexplained variation between GPRNs and practices. We analysed the variations in morbidity estimates by calculating the corresponding median odds ratio (MOR) and 95% confidence intervals (95%CI); we also calculated the odds ratios (ORs) of the significant practice characteristics. MOR quantifies the variation between clusters by comparing two ‘identical’ persons from two randomly chosen, but different clusters. MOR expresses the heterogeneity on an odds ratio scale between clusters and represents the median increased risk. Consequently MOR can never be smaller than one. MOR has been calculated on practice and network level. In this study, MOR refers to the (statistical) increased risk of being diagnosed with a certain disease between two randomly chosen practices or GPRNs. For example, if MOR is 2.0 the risk of being diagnosed with a specific disease is twice as high for a person in one network compared to an ‘identical’ person in another network [19,20].

First, we analysed for each disease the variations in morbidity estimates between general practices and GPRNs without taking any practice characteristic into account. Second, we analysed the influence of six practice characteristics on the variations in morbidity estimates for all diseases in separate models. This results in a total of 154 models (incidence of12 diseases and prevalence of 10 diseases, analysing the variation in one model without any practice characteristics and 6 models with just one practice characteristic (22 × 7 = 154)). Before we performed multilevel analyses, we checked the correlation between characteristics. A high correlation (r >0.70) was found between the urbanization level of the patient’s home address and the distance to the nearest hospital of the general practice. We therefore left urbanization level out of the analyses when measuring the effect of distance to the nearest hospital.

The analyses of type of EHR software package and province could not be performed in a three level analysis, as most GPRNs are located in one province and use only one or two types of EHR software package. The influence of these characteristics was only analysed using LINH data in a two level analysis (patient and practice), since this is the only GPRN located in all provinces and including seven different EHR software packages [21]. All analyses were performed with SAS version 9.2.

## Results

The information of a total of 393,102 patients in 97 practices distributed over six networks was analysed. In total, the participating practices were evenly distributed between solo, duo and group practices. In the different GPNRs, on average 27 to 67 percent of the GPs were female, and the mean number of years of experience of the GPs ranged between 12.3 to 21.3 years. The average distance to the out-of-hours practice or hospital varied between 2.5 and 7 kilometres. As expected, larger distances were seen in more rurally located networks and practices. In general, the mean working experience is higher in networks that exist for a longer period of time. More figures are presented in Table 1.

**Table 1:** **Practice characteristics of six general practice registration networks**

	**Patients** ^**1**^ ***(n)***	**Practice** ^**1**^ ***(n)***	**Type of practice** ^**2**^ ***(n)***			**Practices with POH** ***(%)***	**Female GPs** ***(%)***	**Mean working experience** ***(years)***	**Mean distance (range) to hospital** ^**3**^ ***(km)***	**Mean distance (range) to out-of-hours service location** ^**3**^ ***(km)***
			**Solo**	**duo**	**group**					
ANH VUmc	32 341	7	1	2	4	71.4	62.7	12.7	2.6 (1–4)	2.5 (0–5)
CMR-N	10 291	3	0	1	2	100	41.7	21.3	6.1 (2–9)	7.3 (2–13)
LINH	265 724	72	29	25	18	69.4	27.3	16	7.1 (0–22)	6.1 (0–22)
RNUH Leo	25 263	3	0	0	3	100	44.3	20.1	6.8 (3–12)	4.8 (3–6)
Smile	47 528	8	1	2	5	87.5	66.9	12.2	3.7 (0–7)	3.9 (1–7)
Trans	12 154	4	1	2	1	50	41.8	19.8	6.4 (2–19)	6.4 (2–19)

^1^Total number can deviate from the network population reported elsewhere because incomplete data are excluded. ^2^Based on the number of GPs working in a specific practice. ^3^Estimated on basis of the central position of a postal code, which can be deviated from the actual distance.

As described in the methods section, most GPRNs are located in one province and use extracted data from only one type of EHR software package. In this study, LINH is the only nationally distributed network that was processing data from multiple EHR software packages: Acros, Omnihis, Medicom, Microhis, Mira, Promedico and PromedicoASP. Together these software packages cover more that 80% of the market.

### Influence of practice characteristics on variation between practices and networks

The variations (in MOR) of the 154 models are presented separately for general practices (Additional file 1: Table S1) and GPRNs (Additional file 2: Table S2). In only six cases of the 154 models we observed a significant effect of a practice characteristic on morbidity estimates. Group practices are related to higher estimates of the incidence figures of diabetes mellitus (OR_group_ = 1.74) and anxiety (OR_group_ = 1.54) as compared to solo practices. The prevalence figures of anxiety are negatively related to the distance between the general practice and the out-of-hours service location (OR = 0.96) and hospital (OR = 0.97), for depression this was only the case for the distance between general practice and the out-of-hours service location (OR = 0.96). Furthermore, the employment of a practice nurse leads to higher estimate of the prevalence of COPD (OR = 1.36).

The MOR, for example, of the variation in incidence between general practices of osteoarthritis is 1.42 (95% CI: 1.30-1.55) and the variation in prevalence is 1.60 (95% CI: 1.46-1.65). This means that the chance of being diagnosed with osteoarthritis is respectively 1.4 times higher for incident cases and 1.6 times higher for prevalent cases in one practice compared to another practice. Adding practice characteristics to the estimation of incidence and prevalence rates does not result in lower variations between general practices as MORs remain stable for all health problems (Additional file 1: Table S1).

Considering the variation of osteoarthritis between GPRNs, results show no variation in the incidence rates (MOR 1.02 (95% CI: 1.00-1.45)) and a relatively high variation in prevalence rates (MOR 1.96 (95% CI: 1.48-4.83)). The chance of having a diagnosis of osteoarthritis is about 2 times higher between two randomly chosen GPRNs.

We observed hardly any reductions in the variation between GPRNs after the addition of practice characteristics to the analyses (Additional file 2: Table S2). The same results are seen for most other diseases.

### The influence of EHR software package and province

The influence of EHR software package and province on the variation in incidence and prevalence figures between practices could only be investigated in the LINH network. The effect of EHR software package and province on the variation between practices is small, results are shown in Table 2. Practices using the software package ‘Medicom’ show significantly lower morbidity estimates in 6 out of 10 prevalent disorders. However, this results only in a small decline in variation between practices. For example, in the prevalence of osteoarthritis the MOR between practices decreases from 1.50 (95% CI: 1.38-1.59) to 1.47 (95% CI: 1.37-1.58). For province, practices in “Groningen” showed higher prevalence figures in 3 out of 10 disorders than the other provinces (results not shown), but statistically the variation between practices did not change. For example, in stroke the variance (in MOR) declined from 1.80 (95% CI: 1.60-1.77) to 1.75 (95% CI: 1.56-1.91).

**Table 2:** **The influence of “EHR software package” and “province” on the variation between morbidity estimates of LINH general practices** ^**#**^

**Health problem**	**MOR (95% CI)**				
	***Population characteristics (age, gender, SES, ethnicity and degree of urbanisation***				
	**-**	**EHR software package**		**Province**	
**Incidence**					
Urinary tract infection	-		-		-
Gastro-intestinal infection	-		-		-
Neck and back problems	1.24 (1.17-1.35)		-		-
Eczema	1.27 (1.20-1.40)		-		-
Asthma	1.74 (1.52-2.11)		-		-
COPD	-		-		-
Osteoarthritis	-		-		-
Diabetes Mellitus	1.88 (1.60-2.37)		-		^−^
CHD	2.03 (1.68-2.65)		-	1.86 (1.57-2.40)	Zeeland^1^
Stroke	1.49 (1.30-1.82)		-		-
Depression	1.47 (1.33-1.69)		-		-
Anxiety	1.60 (1.43-1.87)	1.51 (1.36-1.76)	Promedico^1^		-
**Prevalence**					
Neck and back problems	1.33 (1.28-1.40)	1.29 (1.25-1.36)	Medicom^1^		-
Eczema	1.52 (1.44-1.66)	1.50 (1.42-1.63)	Microhis^1^		-
		1.46 (1.39-1.58)	Medicom^1^		
		1.50 (1.42-1.66)	Mira^1^		
Asthma	1.59 (1.50-1.75)	1.57 (1.48-1.71)	Microhis^1^		-
		1.56 (1.47-1.71)	Medicom^1^		
COPD	1.64 (1.49-1.75)		-		-
Osteoarthritis	1.50 (1.38-1.59)	1.47 (1.36-1.56)	Medicom^1^		-
Diabetes Mellitus	1.38 (1.30-1.48)		-	1.36 (1.28-1.45)	Gelderland^1^
CHD	2.03 (1.77-2.23)		-		-
					-
Stroke	1.80 (1.60-1.97)		-	1.75 (1.56-1.91)	Groningen^1^
Depression	1.61 (1.51-1.77)	1.58 (1.48-1.73)	Medicom^1^	1.56 (1.43-1.72)	Groningen^1^
Anxiety	1.71 (1.59-1.90)	1.62 (1.52-1.78)	Microhis^1^	1.67 (1.56-1.85)	Groningen^1^
		1.65 (1.54-1.82)	Medicom^1^		

^#^This table only present the practice characteristics that significantly influenced morbidity estimation on ^1^0.05 level. Note: All variations (in MOR) between general practices are significant in all diseases.

## Discussion

Our results show that only a small number of practice characteristics was related to morbidity estimates. Adjusting for these practice characteristics hardly reduced the variation of morbidity estimates between networks or practices. We did not find any apparent influence of GP or practice characteristics on the variation in morbidity estimates between GPRNs.

Practice characteristics cannot explain the variation between GPRNs or general practices. Still, we found that in group practices more patients were diagnosed with diabetes and anxiety disorders, and practices with a practice nurse showed more patients with the diagnoses COPD. Similar to our findings, in Nielen et al. group practices were associated with higher estimates of incidence of diabetes mellitus (OR = 1.3) [22]. Practice nurses mainly support the GP in monitoring and treating patients with chronic diseases, e.g. diabetes mellitus, CHD, COPD and asthma. A possible explanation for higher prevalence figures of COPD is that a practice nurse with regular contact with these patients keeps better records than the GP.

The relation between psychological problems, such as depression or anxiety, and distance to nearest hospital or out-of-hour service location is probably due to the relationship between large cities and psychological problems. Both psychological problems and smaller distances to a hospital or out-of-hour service locations are more apparent in large cities [23,24]. This is shown in the high correlation found between the urbanization level of patient’s home address and the distance from general practice to the nearest hospital.

To our knowledge, this is the first research that investigates the direct influence of GP and practice characteristics on the variation of morbidity estimates between registration networks, not on the actual morbidity estimation. We explored the practice characteristics that, in earlier research, showed any relevance to morbidity estimation [8-10]. However we must comment that we found particularly small number of significant relations between morbidity figures and practice characteristics. Unfortunately, we could only investigate GP characteristics aggregated on the practice level. This may have diminished the effect of these characteristics on morbidity estimation. Though, research showed that GPs in one practice are more similar than GPs between practices, because a GP’s medical practice is affected by the working environment [8,10,25,26]. Furthermore, Marinus [8] concluded that the investigation of individual GP characteristics on variation would be less effective. Therefore studying the variation at the practice level is legitimate.

A drawback of this study is that we could not differentiate between actual morbidity differences and artefacts of the recording system. Differences between provinces may reflect real differences in health status between populations [27], although there is no reason to expect such large differences of these twelve diseases within a small country as the Netherlands. Overall, no clear effect of province is seen in our data.

Another possible artefact is the type of EHR to record morbidity. Practices using the ‘Medicom’ software package showed lower prevalence figures of osteoarthritis, asthma, eczema, depression, anxiety disorders and neck and back problems. ‘Medicom’ automatically ends an episode if there is no regular contact for this specific health problem. This is often the case in osteoarthritis or stroke. Two GPRNs (SMILE and RNUH Leo) contain only practices, which use the ‘Medicom’ software package, but these GPRNs do not show lower prevalence estimates of osteoarthritis (as we would expect regarding their software package). If a GP marked an episode of osteoarthritis as an episode with special attention, the episode would have stayed active, suggesting a different recording strategy between the different GPRNs.

Other research also showed that inter-doctor variation in morbidity estimates remains high after adjusting for population and practice characteristics [25]. Westert and de Bakker [25] suggested that better use of classification systems by training of GPs might be effective in narrowing the variation. Similar results about the lower number of episodes in practices using ‘Medicom’ compared to other EHR software packages were found by Khan et al. [28]. In their follow up study they observed an increase of the recording quality of the electronic patient records and less variation between practices and between EHR software packages [29].

Variation in morbidity estimates can occur on different stages of the recording process, at the consultation, recording in the EHR, data extraction, data storage, analyses and use of the data for estimation of incidence and prevalence figures. In a previous paper, we investigated the influence of population characteristics on morbidity estimates; in this current research we added the influence of practice characteristics [6]. However, neither population nor practice characteristics could explain the variation between incidence and prevalence estimates between practices or GPRNs. A next step is to investigate the effects of recording agreements of different GPRNs on morbidity variations between GPRNs. The variation between GPRNs is much higher in prevalence figures compared to incidence figures, which might be related to different methods of calculating morbidity [5,26]. For example, some networks only count disease episodes when a patient had contact for that disease in a particular year, as others also include single contacts (not linked to an episode) or episodes with problem status. Understanding the differences between GPRNs and practices is needed to come to the most valid and reliable estimate for morbidity rates in the general population using general practice based data.

## Conclusions

The goal of this study was to explain differences in morbidity estimates from different GPRNs. We investigated to what extent differences in characteristics of general practices could explain this variation. Our results show that only a small number of practice characteristics was related to morbidity estimates. Adjusting for these practice characteristics hardly reduced the variation between networks or practices. Therefore, we conclude that GPs and practice characteristics do not explain the differences in incidence and prevalence figures between different networks.

## Endnote

^a^The name of this network changed in 2013 to NIVEL Primary Care Database (NIVEL-PCD).

## Additional files

Additional file 1: Table S1. The influence of practice characteristics on the variation of incidence and prevalence figures between general practices. Additional file 2: Table S2. The influence of practice characteristics on the variation of incidence and prevalence estimates between general practice registration networks.

## Abbreviations

CHD: Coronary heart disease
COPD: Chronic obstructive pulmonary disease
EHR: Electronic health record
GP: General practitioner
GPRN: General practice registration Network
HAREG: Register of general practitioners of NIVEL
ICD: International classification of disease
ICPC: International classification of primary care
MOR: Median odds ratio
OR: Odds ration
