# Supplementary material for: Do practice characteristics explain differences in morbidity estimates between electronic health record based general practice registration networks?
Source: BMC Fam Pract. 2014 Oct 30;15:176. doi: 10.1186/s12875-014-0176-7 (PMC4231185; doi:10.1186/s12875-014-0176-7)
Supplement: Additional file 1: Table S1. — The influence of practice characteristics on the variation of incidence and prevalence figures between general practices. [file 12875_2014_176_MOESM1_ESM.doc]

**Table S1** **The influence of practice characteristics on the variation of incidence and prevalence figures between general practices**

| **MORBIDITY** | **MOR (95%CI)** | | | | | | |
| --- | --- | --- | --- | --- | --- | --- | --- |
|  | **Population characteristics (age, gender, SES, ethnicity and degree of urbanisation)** | | | | | | |
|  | **-** | **Type of practice** | **%female** | **Working experience** | **Practice nurse** | **Distance to hospital^1^** | **Distance to out-of-hours service location** |
| **Incidence** | | | | | | | |
| Urinary tract infection | **1.33** | **1.32** | **1.31** | **1.34** | **1.32** | **1.34** | **1.33** |
|  | 1.25-1.47 | 1.24-1.46 | 1.24-1.40 | 1.25-1.48 | 1.24-1.46 | 1.25-1.48 | 1.25-1.48 |
| Gastro-intestinal infection | **1.52** | **1.50** | **1.52** | **1.53** | **1.53** | **1.53** | **1.53** |
|  | 1.39-1.67 | 1.37-1.66 | 1.38-1.67 | 1.39-1.69 | 1.39-1.69 | 1.39-1.68 | 1.39-1.69 |
| Neck and back problems | **1.23** | **1.23** | **1.22** | **1.23** | **1.23** | **1.22** | **1.23** |
|  | 1.17-1.32 | 1.17-1.32 | 1.17-1.32 | 1.17-1.32 | 1.18-1.32 | 1.17-1.31 | 1.18-1.32 |
| Eczema | **1.29** | **1.28** | **1.28** | **1.27** | **1.28** | **1.28** | **1.29** |
|  | 1.22-1.40 | 1.22-1.40 | 1.22-1.40 | 1.21-1.38 | 1.22-1.40 | 1.22-1.40 | 1.22-1.40 |
| Asthma | **1.73** | **1.75** | **1.70** | **1.73** | **1.73** | **1.72** | **1.72** |
|  | 1.53-1.95 | 1.55-1.99 | 1.51-1.92 | 1.53-1.96 | 1.54-1.96 | 1.53-1.95 | 1.52-1.94 |
| COPD | **1.42** | **1.41** | **1.41** | **1.41** | **1.39** | **1.44** | **1.43** |
|  | 1.28-1.60 | 1.27-1.61 | 1.27-1.61 | 1.27-1.60 | 1.28-1.57 | 1.30-1.63 | 1.28-1.62 |
| Osteo-arthritis | **1.42** | **1.43** | **1.42** | **1.41** | **1.43** | **1.41** | **1.42** |
|  | 1.30-1.55 | 1.31-1.57 | 1.30-1.55 | 1.29-1.55 | 1.31-1.57 | 1.30-1.54 | 1.30-1.55 |
| Diabetes Mellitus | **1.65** | **1.58** | **1.61** | **1.63** | **1.65** | **1.65** | **1.66** |
|  | 1.47-1.85 | 1.42-1.79 | 1.44-1.82 | 1.46-1.84 | 1.47-1.86 | 1.48-1.86 | 1.48-1.87 |
| CHD | **1.65** | **1.63** | **1.63** | **1.66** | **1.65** | **1.65** | **1.65** |
|  | 1.46-1.88 | 1.45-1.87 | 1.45-1.86 | 1.47-1.90 | 1.47-1.89 | 1.47-1.89 | 1.46-1.89 |
| Stroke | **1.36** | **1.34** | **1.37** | **1.37** | **1.37** | **1.37** | **1.36** |
|  | 1.24-1.53 | 1.19-1.52 | 1.23-1.54 | 1.24-1.54 | 1.23-1.54 | 1.24-1.54 | 1.22-1.54 |
| Depression | **1.46** | **1.44** | **1.45** | **1.47** | **1.47** | **1.44** | **1.46** |
|  | 1.34-1.62 | 1.32-1.59 | 1.33-1.61 | 1.34-1.64 | 1.35-1.64 | 1.32-1.60 | 1.34-1.63 |
| Anxiety | **1.54** | **1.50** | **1.46** | **1.55** | **1.54** | **1.55** | **1.55** |
|  | 1.40-1.71 | 1.37-1.66 | 1.34-1.61 | 1.41-1.72 | 1.40-1.71 | 1.41-1.72 | 1.41-1.72 |
| **Prevalence** | | | | | | | |
| Neck and back problems | **1.41** | **1.40** | **1.41** | **1.41** | **1.41** | **1.41** | **1.41** |
|  | 1.36-1.49 | 1.36-1.48 | 1.37-1.49 | 1.37-1.49 | 1.36-1.49 | 1.37-1.49 | 1.36-1.49 |
| Eczema | **1.60** | **1.60** | **1.60** | **1.60** | **1.60** | **1.60** | **1.58** |
|  | 1.52-1.72 | 1.52-1.72 | 1.53-1.72 | 1.53-1.73 | 1.53-1.73 | 1.53-1.73 | 1.51-1.70 |
| Asthma | **1.65** | **1.66** | **1.65** | **1.65** | **1.64** | **1.65** | **1.64** |
|  | 1.57-1.73 | 1.57-1.74 | 1.57-1.73 | 1.57-1.73 | 1.56-1.72 | 1.57-1.73 | 1.56-1.72 |
| COPD | **1.68** | **1.69** | **1.68** | **1.68** | **1.66** | **1.68** | **1.68** |
|  | 1.59-1.77 | 1.53-1.78 | 1.52-1.77 | 1.52-1.78 | 1.51-1.75 | 1.59-1.77 | 1.59-1.77 |
| Osteo-arthritis | **1.60** | **1.59** | **1.60** | **1.60** | **1.59** | **1.60** | **1.60** |
|  | 1.46-1.68 | 1.45-1.67 | 1.46-1.68 | 1.46-1.68 | 1.46-1.67 | 1.46-1.68 | 1.46-1.68 |
| Diabetes Mellitus | **1.52** | **1.53** | **1.53** | **1.53** | **1.51** | **1.52** | **1.53** |
|  | 1.46-1.59 | 1.46-1.59 | 1.46-1.59 | 1.56-1.59 | 1.45-1.58 | 1.46-1.59 | 1.46-1.59 |
| CHD | **1.91** | **1.90** | **1.89** | **1.91** | **1.90** | **1.92** | **1.90** |
|  | 1.78-2.03 | 1.78-2.03 | 1.77-2.02 | 1.79-2.04 | 1.77-2.03 | 1.79-2.04 | 1.77-2.03 |
| Stroke | **1.76** | **1.77** | **1.77** | **1.77** | **1.77** | **1.75** | **1.77** |
|  | 1.73-1.88 | 1.73-1.89 | 1.73-1.89 | 1.73-1.89 | 1.73-1.89 | 1.71-1.86 | 1.73-1.89 |
| Depression | **1.71** | **1.72** | **1.71** | **1.71** | **1.71** | **1.71** | **1.71** |
|  | 1.62-1.80 | 1.62-1.81 | 1.62-1.81 | 1.62-1.81 | 1.62-1.81 | 1.62-1.80 | 1.59-1.77 |
| Anxiety | **1.74** | **1.75** | **1.73** | **1.74** | **1.74** | **1.74** | **1.71** |
|  | 1.64-1.83 | 1.64-1.85 | 1.64-1.85 | 1.64-1.83 | 1.65-1.84 | 1.64-1.83 | 1.62-1.80 |

**Bold:** all differences between practices are significant. Shaded cells represent a significant influence of the specific practice characteristic on morbidity estimation (p < 0.05), corresponding odds ratio is not reported. ^1^Level of urbanisation of the home address of the patient is not considered, because of high correlation to distance to hospital.
