# Supplementary material for: Do practice characteristics explain differences in morbidity estimates between electronic health record based general practice registration networks?
Source: BMC Fam Pract. 2014 Oct 30;15:176. doi: 10.1186/s12875-014-0176-7 (PMC4231185; doi:10.1186/s12875-014-0176-7)
Supplement: Additional file 2: Table S2. — The influence of practice characteristics on the variation of incidence and prevalence estimates between general practice registration networks. [file 12875_2014_176_MOESM2_ESM.doc]

**Table S2** **The influence of practice characteristics on the variation of incidence and prevalence estimates between general practice registration networks**

| **MORBIDITY** | **MOR (95%CI)** | | | | | | |
| --- | --- | --- | --- | --- | --- | --- | --- |
|  | **Population characteristics (age, gender, SES, ethnicity and degree of urbanisation)** | | | | | | |
|  | **-** | **Type of practice** | **%female** | **Working experience** | **Practice nurse** | **Distance to hospital^1^** | **Distance to out-of-hours service location** |
| **Incidence** | | | | | | | |
| Urinary tract infection | 1.18 | 1.19 | 1.20 | 1.12 | 1.19 | 1.18 | 1.18 |
|  | 1.00-1.66 | 1.00-1.69 | 1.00-1.71 | 1.00-1.55 | 1.00-1.68 | 1.00-1.66 | 1.00-1.68 |
| Gastro-intestinal infection | **1.44** | **1.43** | **1.41** | **1.44** | **1.44** | **1.45** | **1.44** |
|  | 1.20-2.66 | 1.18-2.64 | 1.16-2.53 | 1.19-2.66 | 1.19-2.66 | 1.20-2.67 | 1.20-2.66 |
| Neck and back problems | **1.29** | **1.28** | **1.28** | **1.29** | **1.29** | **1.29** | **1.29** |
|  | 1.12-1.98 | 1.11-1.95 | 1.11-1.95 | 1.13-1.98 | 1.12-1.99 | 1.13-1.98 | 1.12-1.98 |
| Eczema | 1.18 | 1.17 | 1.18 | **1.24** | 1.16 | 1.17 | 1.17 |
|  | 1.00-1.66 | 1.00-1.66 | 1.00-1.68 | 1.05-1.84 | 1.00-1.62 | 1.00-1.65 | 1.00-1.65 |
| Asthma | **1.39** | **1.37** | **1.47** | **1.38** | **1.41** | **1.39** | **1.38** |
|  | 1.09-2.71 | 1.06-2.69 | 1.15-2.97 | 1.08-2.68 | 1.10-2.86 | 1.09-2.72 | 1.09-2.86 |
| COPD | **1.40** | **1.48** | **1.45** | **1.40** | **1.49** | **1.42** | **1.40** |
|  | 1.13-2.69 | 1.16-3.04 | 1.16-2.82 | 1.13-2.64 | 1.19-3.05 | 1.13-2.82 | 1.13-2.69 |
| Osteo-arthritis | 1.02 | 1.02 | 1.00 | 1.03 | 1.03 | 1.08 | 1.02 |
|  | 1.00-1.42 | 1.00-1.44 | 1.00-1.52 | 1.00-1.44 | 1.00-1.44 | 1.00-1.48 | 1.00-1.42 |
| Diabetes Mellitus | 1.00 | 1.14 | 1.15 | 1.00 | 1.00 | 1.00 | 1.00 |
|  | 1.00-1.45 | 1.00-1.71 | 1.00-1.74 | 1.00-1.58 | 1.00-1.61 | 1.00-1.55 | 1.00-1.56 |
| CHD | 1.00 | 1.00 | 1.00 | 1.00 | 1.00 | 1.00 | 1.00 |
|  | 1.00-1.42 | 1.00-1.71 | 1.00-1.60 | 1.00-1.60 | 1.00-1.58 | 1.00-1.56 | 1.00-1.56 |
| Stroke | 1.20 | 1.27 | 1.19 | 1.17 | 1.21 | 1.21 | 1.21 |
|  | 1.00-1.88 | 1.00-2.14 | 1.00-1.87 | 1.00-1.80 | 1.00-1.93 | 1.00-1.91 | 1.00-1.91 |
| Depression | 1.38 | **1.51** | 1.41 | 1.38 | 1.38 | **1.48** | 1.39 |
|  | 1.00-2.79 | 1.10-3.24 | 1.00-2.76 | 1.00-2.77 | 1.00-2.80 | 1.06-3.11 | 1.00-2.79 |
| Anxiety | **1.66** | **1.78** | **1.73** | **1.66** | **1.70** | **1.75** | **1.65** |
|  | 1.17-4.07 | 1.29-4.72 | 1.29-4.30 | 1.17-4.09 | 1.20-4.30 | 1.23-4.55 | 1.17-4.06 |
| **Prevalence** | | | | | | | |
| Neck and back problems | **1.90** | **1.92** | **1.91** | **1.90** | **1.90** | **1.90** | **1.89** |
|  | 1.46-4.38 | 1.47-4.47 | 1.47-4.42 | 1.46-4.37 | 1.47-4.40 | 1.47-4.40 | 1.46-4.33 |
| Eczema | **1.57** | **1.59** | **1.55** | **1.57** | **1.57** | **1.56** | **1.57** |
|  | 1.29-2.91 | 1.30-2.99 | 1.27-2.83 | 1.29-2.92 | 1.29-2.93 | 1.28-2.86 | 1.29-2.94 |
| Asthma | **1.37** | **1.37** | **1.36** | **1.37** | **1.37** | **1.38** | **1.38** |
|  | 1.11-2.20 | 1.11-2.21 | 1.10-2.20 | 1.11-2.21 | 1.11-2.21 | 1.11-2.24 | 1.12-2.24 |
| COPD | **1.46** | **1.44** | **1.44** | **1.46** | **1.42** | **1.46** | **1.46** |
|  | 1.18-2.55 | 1.16-2.52 | 1.14-2.49 | 1.17-2.55 | 1.15-2.42 | 1.18-2.57 | 1.18-2.59 |
| Osteo-arthritis | **1.96** | **2.00** | **1.97** | **1.98** | **1.99** | **1.99** | **1.96** |
|  | 1.48-4.83 | 1.50-5.04 | 1.48-4.87 | 1.48-4.92 | 1.49-5.00 | 1.49-4.99 | 1.48-4.85 |
| Diabetes Mellitus | **1.34** | **1.35** | **1.34** | **1.34** | **1.33** | **1.34** | **1.34** |
|  | 1.11-2.08 | 1.12-2.12 | 1.11-2.09 | 1.11-2.09 | 1.11-2.05 | 1.11-2.08 | 1.11-2.08 |
| CHD | **2.38** | **2.40** | **2.34** | **2.39** | **2.38** | **2.37** | **2.43** |
|  | 1.65-7.69 | 1.65-7.77 | 1.63-7.42 | 1.65-7.74 | 1.64-.63 | 1.64-7.60 | 1.67-8.03 |
| Stroke | **2.20** | **2.21** | **2.19** | **2.20** | **2.19** | **2.18** | **2.23** |
|  | 1.57-6.36 | 1.58-6.47 | 1.57-6.33 | 1.57-6.37 | 1.57-6.33 | 1.56-6.25 | 1.58-6.57 |
| Depression | **1.58** | **1.59** | **1.56** | **1.59** | **1.59** | **1.58** | **1.58** |
|  | 1.27-3.06 | 1.27-3.10 | 1.24-2.98 | 1.27-3.08 | 1.27-3.09 | 1.26-3.07 | 1.27-3.03 |
| Anxiety | **1.50** | **1.51** | **1.47** | **1.52** | **1.50** | **1.50** | **1.51** |
|  | 1.19-2.75 | 1.20-2.80 | 1.15-2.63 | 1.20-2.82 | 1.19-2.76 | 1.19-2.78 | 1.20-2.77 |

**Bold:** Significant variation between GPRNs. Shaded cells represent a significant influence of the specific practice characteristic on morbidity estimation (p < 0.05), corresponding odds ratio is not reported. ^1^Level of urbanisation of the home address of the patient is not considered, because of high correlation to distance to hospital.
